# Updated survivals and prognostic factor analysis in myeloma treated by a staged approach use of bortezomib/thalidomide/dexamethasone in transplant eligible patients

**DOI:** 10.1186/1479-5876-8-124

**Published:** 2010-11-26

**Authors:** Chor Sang Chim

**Affiliations:** 1 Department of Medicine, Queen Mary Hospital, University of Hong Kong, Hong Kong

## Abstract

**Background:**

Bortezomib, an NFkB inhibitor, is an active agent for the treatment of myeloma (MM). We have reported a promising complete remission (CR) rate for newly diagnosed myeloma patients treated by a staged approach, in which chemosensitive patients underwent autologous haematopoietic stem cell transplantation (auto-HSCT) while less chemosensitive patients received salvage therapy with bortezomib/thalidomide/dexamethasone prior to auto-HSCT.

**Methods:**

Herein, with an additional 13 months of follow-up, we reported the updated survivals, and examined potential prognostic factors impacting event-free (EFS) and overall survival (OS).

**Results:**

With a median follow-up of 30 months, the projected OS was 73% and EFS was 50.2%. Age, gender, clinical stage and DAPK methylation could not account for the differential chemosensitivity. Advanced ISS stage and DAPK methylation adversely impacted OS whereas oligoclonal reconstitution predicted superior EFS.

**Conclusions:**

Our staged approach illustrated an economical use of expensive targeted agents while preserving a good CR rate and OS. The comparable survivals of chemosensitive and less chemosensitive patients suggested the staged approach might have abolished the adverse prognostic impact of suboptimal chemosensitivity. Finally, the adverse impact of DAPK methylation and favorable impact of oligoclonal reconstitution in myeloma warrants further study.

## Background

Bortezomib, an NFkB inhibitor, is an active agent for the treatment of myeloma (MM). After the demonstration of its efficacy as salvage therapy in chemo-resistant or refractory myeloma patients with a CR rate of 9% [1,2]. a high CR rate has also been demonstrated when bortezomib was used in induction therapy in newly diagnosed myeloma patients. For instance, a CR rate of 43% and 30% was observed when bortezomib-based induction therapy was applied in both transplant-eligible and transplant-ineligible myeloma patients [3,4].

**Figure 1:**
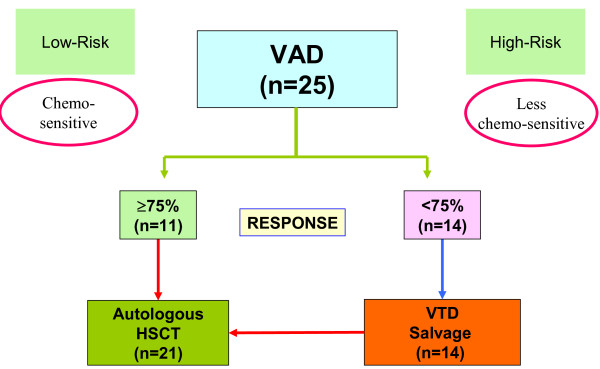
**Treatment algorithm of the staged approach for newly diagnosed, symptomatic myeloma patients**.

In Hong Kong, we have adopted a staged approach, in which newly diagnosed, transplant-eligible myeloma patients were risk-stratified according to their initial chemosensitivity, wherein VAD-chemosensitive patients underwent autologous hematopoietic stem cell transplantation (auto-HSCT) while less VAD-chemosensitive patients received salvage therapy of bortezomib/thalidomide/dexamethasone (VTD) before auto-HSCT.^5 ^(Figure 1) We have reported frequent occurrence of oligoclonal reconstitution, frequent central nervous system myeloma (one with leptomeningeal myeloma presenting with diplopia, and the other with intraspinal plasmacytoma causing spinal cord compression) and absence of thalidomide-related deep-vein thrombosis despite no prophylaxis with either aspirin, low molecular weight heparin or warfarin [5]. In addition, at a median follow-up time of 17 months, we have reported an overall CR rate of 48% (by an intention-to-treat analysis), and a 3-year OS and 75% [5]. Based on this approach, only 56% myeloma patients required salvage therapy with VTD. Herein, with an extended follow-up (median: 30 months, range: 7-54 months), we reported the updated survivals. In particular, we examined if diagnostic clinical parameters might account for the differential VAD chemosensitivity. Moreover, potential risk factors for EFS and OS, including methylation of Death-associated Protein Kinase (*DAPK*) and the development of oligoclonal reconstitution, were analysed.

## Methods

### Treatment

The study started in early 2005 and ended in late 2008. The median follow-up time was 30 months (range: 7 - 54 months). Details of the trial has been reported [5]. In brief, 25 newly diagnosed, symptomatic MM with younger than 65 years with measurable disease were enrolled. All patients received initial cytoreduction with three cycles of VAD (vincristine, adriamycin and dexamethasone). Those achieving ≥ 75% reduction in paraprotein, i.e. VAD-chemosensitive patients, proceeded to auto-HSCT. Patients with <75% reduction in paraprotein, i.e. less chemosensitive subgroup, received salvage therapy with four cycles of VTD (bortezomib: 1.3 mg/m^2^/day intravenously on days 1, 4, 8 and 11; thalidomide: 200 mg/day; dexamethasone: 40 mg/d orally from days 1-4 and days 8-11). After VAD induction therapy, fourteen (56%) patients required VTD salvage therapy. Auto-HSCT conditioning regimen comprised intravenous melphalan at 200 mg/m^2^. All patients received thalidomide (100-200 mg/day) as maintenance therapy regardless of whether VTD had been used. The protocol was approved by the institution review board in accordance with the Declaration of Helsinki, and informed consent was obtained from all participating patients. The treatment algorithm was shown in Figure 1.

### Monitoring of response

All patients were analyzed on an intention-to-treat basis. Progression was defined as ≥25% paraprotein increase in two consecutive tests four weeks apart. Relapse was defined as reappearance of the paraprotein on immunofixation in CR patients, positive SPE in the nCR patients, and/or appearance of new bone lesions. Oligoclonal reconstitution, defined as the appearance of a new paraprotein persisting for ≥ 4 weeks, was demonstrated in six patients [5]. Three patients with light chain myeloma developed a IgG paraprotein (two IgG/kappa from free kappa, one IgG/lambda from free lambda). One developed a double IgG/kappa from a single IgG/kappa, and two patients had complete change of paraprotein (one from IgA/kappa to IgG kappa, and one from IgD/lambda to IgG/kappa).

### Statistical analysis

OS was defined as time from commencement of induction therapy to death or last follow-up. Event-free survival (EFS) was defined as time from commencement of induction therapy to the date of progression, relapse or death. Survival curves were plotted by Kaplan-Meier method. Prognostic factors including age, gender, international staging system (ISS) [6]. DAPK methylation status and achievement of CR after auto-HSCT were studied for their impact on survival by univariate analysis. Survival curves were plotted by Kaplan-Meier method and compared by the log-rank test [7]. Moreover, to see if early PR after one cycle of VAD might predict subsequent need of VTD salvage therapy, achievement of PR, i.e >50% reduction in paraprotein, after one cycle of VAD was correlated with subsequent need of VTD salvage upon completion of three cycles of VAD by Chi-Square test.

### Methylation study

Methylation-specific polymerase chain reaction (MSP) for aberrant promoter methylation was performed as previously described [8-10]. Treatment of DNA with bisulphite for conversion of unmethylated cytosine to uracil (but unaffecting methylated cytosine) was performed with a commercially available kit (CpGenome DNA modification kit, Intergen, New York). The primers for the methylated (M-MSP) and unmethylated (U-MSP) promoters of *DAPK *has been previously described [10-12].

## Results

**Figure 2:**
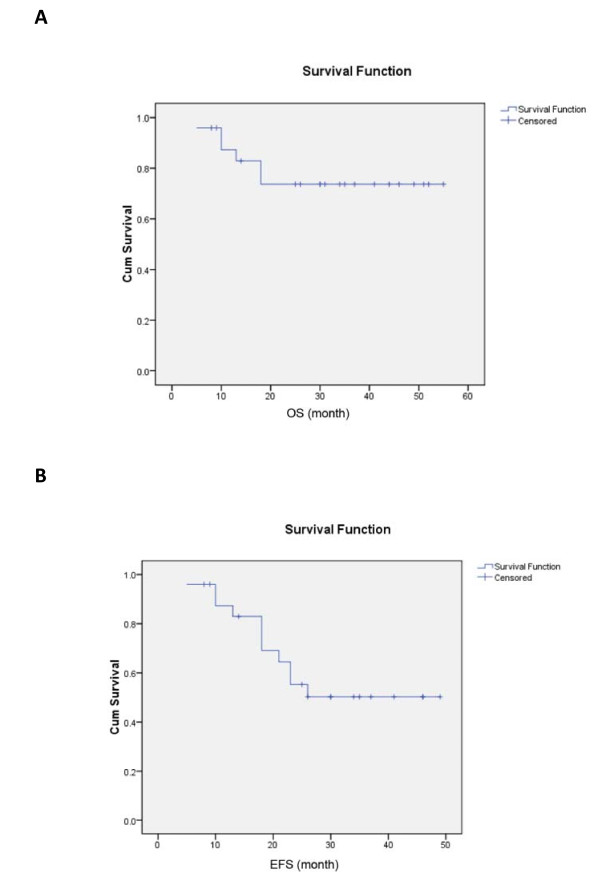
**(A) Updated Overall survival (OS) and (B) Event-free survival (EFS) for the whole group**.

The projected 4-year OS was 73.7%, the 4-year EFS was 50.2% with a median follow-up time of 30 months. (Figure 2)

**Figure 3:**
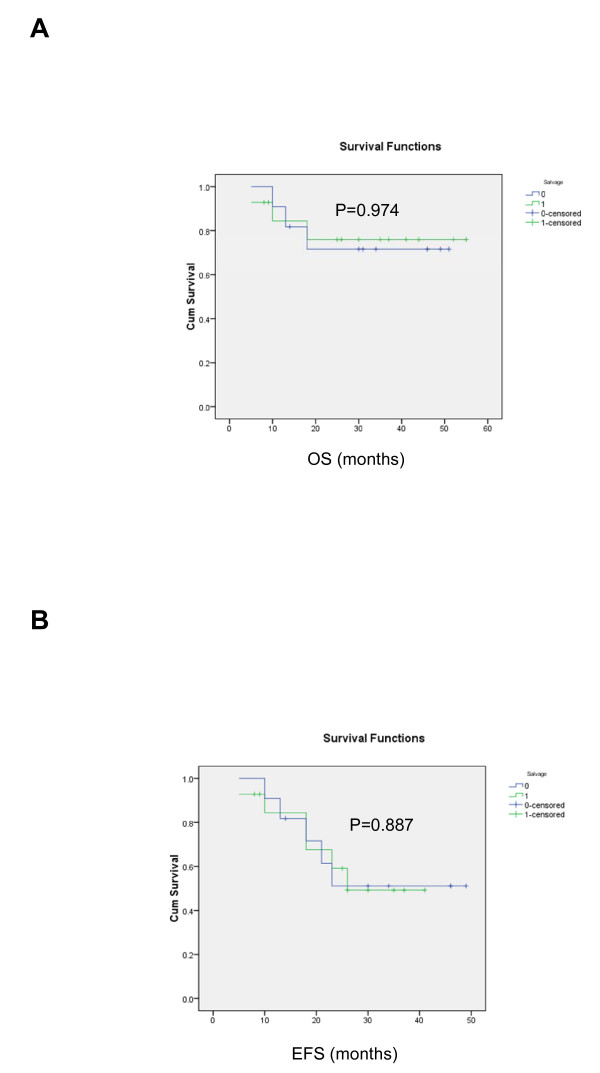
**(A) OS and (B) EFS of VAD-chemosensitive (green line) and less chemosensitive (blue line) patients, showing comparable OS and EFS in VAD-chemosensitive and less chemosensitive patients**.

Comparing the VAD-chemosensitive with the less chemosensitive subgroups, there was no significant difference in the median age and distribution of gender, paraprotein subtypes and International Stage. (Table 1) Of the 22 patients with methylation study data, four (18.2%) carried *DAPK *methylation. However, the proportion of patients carrying *DAPK *methylation or developing oligoclonal reconstitution was not different. On the other hand, more chemosensitive patients (90.9%) achieved ≥VGPR after auto-HSCT than those less chemosensitive patients (p = 0.03). The projected EFS were 51.1% for chemosensitive, and 49.2% for less chemosensitive patients (p = 0.974)(Table 2). The projected OS was 71.6% and 76.0% for chemosensitive and less chemosensitive patients (p = 0.887) (Figure 3) (Table 2).

**Table 1:** Differences between VAD-chemosensitive (CS) and less VAD-chemosensitive (LCS) patients

	CS [%]	LCS [%]	P-value
Gender			0.99
Male	7 [63.6]	10 [71.4]	
female	4 [36.4]	4 [28.6]	
Age (median)	49	55	0.217
paraprotein subtype			0.07
G	3 [27.3]	9 [64.3]	
A	1 [9.1]	3 [21.4]	
D	2 [18.2]	0 [0]	
LC	5 [45.5]	2 [14.3]	
ISS stage			0.69
I & II	7 [63.6]	7 [50]	
III	4 [36.4]	7 [50]	
≥VGPR after induction			0.227
No	3 [27.3]	8 [57.1]	
Yes	8 [72.7]	6 [42.9]	
≥VGPR after ABMT			0.03
No	1 [9.1]	7 [50.0]	
Yes	10 [90.9]	7 [50.0]	
Oligoclonal reconstitution			0.434
Yes	5 [45.5]	4 [28.6]	
no	6 [54.5]	10 [71.4]	
DAPK methylation			0.216
methylated	3 [27.3]	1 [7.1]	
unmethylated	6 [54.5]	12 [85.7]	
unknown	2 [18.2]	1 [7.1]1	

G: IgG; A: IgA; D: IgD; LC: light chain myeloma; DS: Durie-Salmon stage; ISS: International staging system; VGPR: >90% reduction in paraprotein level

**Table 2:** P-values for univariate analysis of prognostic factors

	OS	EFS
Gender	0.081	0.211
Age (median)	0.828	0.770
Paraprotein subtype	0.382	0.393
ISS	0.026	0.645
VAD chemosensitivity	0.887	0.974
VGPR after induction	0.722	0.406
VGPR after auto-HSCT	0.181	0.357
Oligoclonal reconstitution	0.170	0.039
DAPK methylation	0.029	0.136

DS: Durie-Salmon stage; ISS: International staging system; VGPR: >90% reduction in paraprotein level; OS: overall survival; EFS: event-free survival

Of the 23 patients with response data after one cycle of VAD, 11 (48.8%) fail to achieve PR. Of these, 10 (90.9%) finally required VTD salvage therapy as the culmulative response after three cycles of VAD was <75% paraprotein reduction. (p < 0.001)

**Figure 4:**
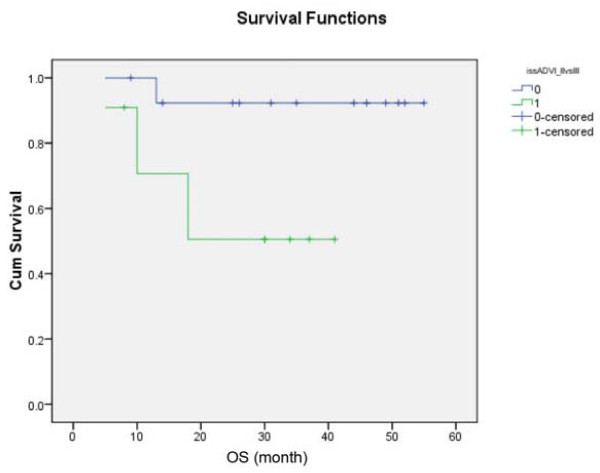
**Impact of advanced (green line) and limited ISS stage (blue line) on OS, showing inferior survival in patients with advanced ISS stage**.

**Figure 5:**
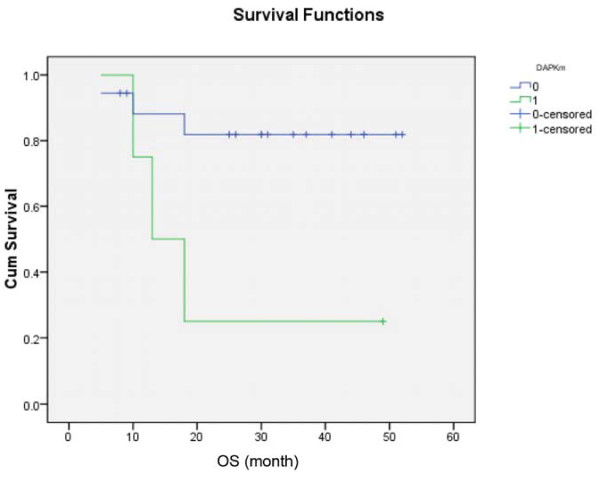
**Impact of the presence (green line) and absence of (blue line) DAPK methylation on OS, showing inferior survival in patients with DAPK methylation**.

**Figure 6:**
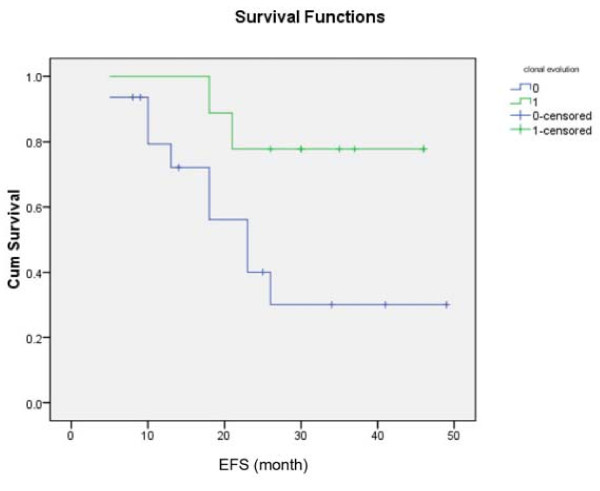
**Impact of the absence (blue line) and presence (green line) of oligoclonal reconstitution on EFS, showing a superior EFS in patients with oligoclonal reconstitution**.

Analysis of risk factors for survivals showed that only advanced ISS (0.034) (Figure 4) and *DAPK *methylation (p = 0.02) (Figure 5) predicted inferior OS but not EFS. On the other hand, development of oligoclonal reconstitution predicted superior EFS but not OS. (Figure 6) However, age, gender, VAD-sensitivity, attainment of ≥VGPR after induction therapy and achievement of ≥VGPR after auto-HSCT did not impact either EFS or OS. (Table 2)

## Discussion

This extended follow-up study revealed a EFS and an OS comparable to another study using bortezomib/adriamcyin/dexamethasone (PAD) regimen as frontline therapy in newly diagnosed myeloma, in which the median EFS was 29 months, and the 4-year OS was 73% [3]. However, in our study, only 56% patients required the use of bortezomib-based salvage therapy. Therefore, this staged approach will carry significant impact on healthcare financing systems in less affluent countries. For instance, had all our patients been treated with four cycles of VTD upfront prior to auto-HSCT (with four injections of bortezomib on days 1, 4, 8 and 11 in each cycle, costing USD4800 per cycle), then an additional 11 patients (i.e. those failing to achieve ≥ 75% reduction in paraprotein level) would have required four cycles of bortezomib, and hence an additional cost of USD211,200.

Moreover, to see if early PR after one cycle of VAD may predict subsequent need of VTD salvage therapy, achievement of PR (i.e >50% reduction in paraprotein) after one cycle of VAD was correlated with subsequent need of VTD salvage after three cycles of VAD by Chi-Square test. Of the 23 patients with response data after one cycle of VAD, 11 (48.8%) fail to achieve PR. Of these, 10 (90.9%) finally required VTD salvage therapy as the culmulative response after three cycles of VAD was <75% paraprotein reduction. (p < 0.001) Therefore, patients who failed to achieve >75% paraprotein reduction, and hence ultimately require VTD salvage, could in fact be predicted by the ability to achievev a PR after one cycle of VAD, which would reduce the incidence or severity of sensory neuropathy associated with the subsequent use of VTD.

Our approach was based on risk-stratification by initial VAD-chemosensitivity. Therefore, we studied if the differential VAD-chemosensitivity might be associated with favorable risk factors, and hence attributable to the clinical parameters including age, gender, DS and ISS stage. However, no difference was demonstrated in the distribution of these risk factors in the chemosensitive and less chemosensitive patients. On the other hand, DNA methylation may be an important biomarker [7,13-15]. In particular, methylation of *DAPK*, a tumor suppressor gene, has been analyzed. However, there was no difference in the proportion of patients with *DAPK *methylation. Therefore, neither clinical parameters nor *DAPK *methylation could account for the differential VAD chemosensitivity.

As chemosensitivity is an important risk factor for survival, we postulated that the higher chemosensitivity might indeed translate into superior EFS and OS. However, there was no difference in the median EFS and OS between the chemosensitive and less chemosensitive subgroups, implying that the potential adverse prognostic impact of suboptimal chemosensitivity has been abolished by salvage therapy with the VTD regimen.

In an attempt to study the potential clinical risk factors for survival, parameters including age, gender, DS stage and ISS were analyzed for their impact on survival. Moreover, achievement of VGPR both prior to or after auto-HSCT have been shown to predict superior EFS and OS [16], and hence have been analyzed for their impact on survivals. Amongst these factors, only advanced ISS and *DAPK *methylation were the risk factors predicting inferior OS. While *DAPK *methylation has been shown to predict inferior survival in retrospective analyses, those myeloma patients have received heterogeneous treatments in those studies [10]. By contrast, patients were uniformly treated in this study. However, the number of patients with *DAPK *methylation data was small, and hence the statistical power of the analysis was diminished. Therefore, the role of *DAPK *methylation in myeloma warrants further study with larger number of patients treated in a uniform manner. Moreover, current data have shown that cytogenetic study is an important biological risk factor. For instance, del(17p), t(4;14) and t(14;16) are high-risk karyotypic aberrations predicting inferior survivals, which might be reversed by the frontline use of bortezomib-containing induction regimens such as Bortezomib-melphalan-prednisolone (VMP) or bortezomib-dexamethasone [17,18]. Therefore, ideally, karyotypic data, not available in our patients, should be included into the study.

Finally, recent studies showed that oligoclonal reconstitution was associated with a higher response rate and CR rate, thereby predicting robust response [19] Indeed, in this study, oligoclonal reconstitution predicted superior EFS but not OS, which is due to the successful salvage therapy. Therefore, this is the first report of superior EFS associated with the development of oligoclonal reconstitution.

## Conclusions

Our staged approach yielded survivals comparable to studies using bortezomib-based regimens as induction therapy, thereby limiting the use of bortezomib-based salvage to about half of the patients without adversely affecting treatment outcome.

The comparable survivals of chemosensitive and less chemosensitive patients suggested the staged approach might have abolished the adverse prognostic impact of suboptimal chemosensitivity. In view of the promising results from this study, the staged approach will be adopted for treatment of transplant-eligible myeloma patients in the future in Hong Kong except that thalidomide/dexamethasone will be used instead of VAD.

Finally, *DAPK *methylation and oligoclonal reconstitution as potential adverse and favorable risk factors in myeloma warrants further validation with larger number of patients in prospective clinical trials. These might prove to be useful prognostic factors in addition to chromosomal aberrations and the International Staging System.

## Competing interests

The author declares that they have no competing interests.

## Authors' contributions

CSC is responsible for the conception, design, and acquisition of data, analysis and interpretation of data, writing and approval of the manuscript.
